# Theoretical basis of the community effect in development

**DOI:** 10.1186/1752-0509-5-54

**Published:** 2011-04-17

**Authors:** Yasushi Saka, Cédric Lhoussaine, Celine Kuttler, Ekkehard Ullner, Marco Thiel

**Affiliations:** 1 School of Medical Sciences, Institute of Medical Sciences, University of Aberdeen, Foresterhill, Aberdeen AB25 2ZD, UK; 2 LIFL, UMR Université Lille 1/CNRS 8022, Cité Scientifique, Bat M3, 59655 Villeneuve d'Ascq, Cedex France; 3 Interdisciplinary Research Institute, CNRS USR3078, Parc de la Haute Borne, 50 avenue Halley, BP70478, 59658 Villeneuve d'Ascq, France; 4 Department of Physics, Institute for Complex Systems and Mathematical Biology, SUPA, University of Aberdeen, Old Aberdeen, Aberdeen AB24 3UE, UK

## Abstract

**Background:**

Genetically identical cells often show significant variation in gene expression profile and behaviour even in the same physiological condition. Notably, embryonic cells destined to the same tissue maintain a uniform transcriptional regulatory state and form a homogeneous cell group. One mechanism to keep the homogeneity within embryonic tissues is the so-called community effect in animal development. The community effect is an interaction among a group of many nearby precursor cells, and is necessary for them to maintain tissue-specific gene expression and differentiate in a coordinated manner. Although it has been shown that the cell-cell communication by a diffusible factor plays a crucial role, it is not immediately obvious why a community effect needs many cells.

**Results:**

In this work, we propose a model of the community effect in development, which consists in a linear gene cascade and cell-cell communication. We examined the properties of the model theoretically using a combination of stochastic and deterministic modelling methods. We have derived the analytical formula for the threshold size of a cell population that is necessary for a community effect, which is in good agreement with stochastic simulation results.

**Conclusions:**

Our theoretical analysis indicates that a simple model with a linear gene cascade and cell-cell communication is sufficient to reproduce the community effect in development. The model explains why a community needs many cells. It suggests that the community's long-term behaviour is independent of the initial induction level, although the initiation of a community effect requires a sufficient amount of inducing signal. The mechanism of the community effect revealed by our theoretical analysis is analogous to that of quorum sensing in bacteria. The community effect may underlie the size control in animal development and also the genesis of autosomal dominant diseases including tumorigenesis.

## Background

During embryonic development, cell-cell interaction plays a pivotal role in generating many types of cells that constitute a functional adult body. The most prevalent of such interaction is embryonic induction, a process by which part of a tissue within the embryo changes its direction of differentiation into another upon receipt of a signal emanating from the nearby tissue. Such induction events, however, are transient and therefore the cells that have received the signal must 'remember' the event until they terminally differentiate.

**Figure 1:**
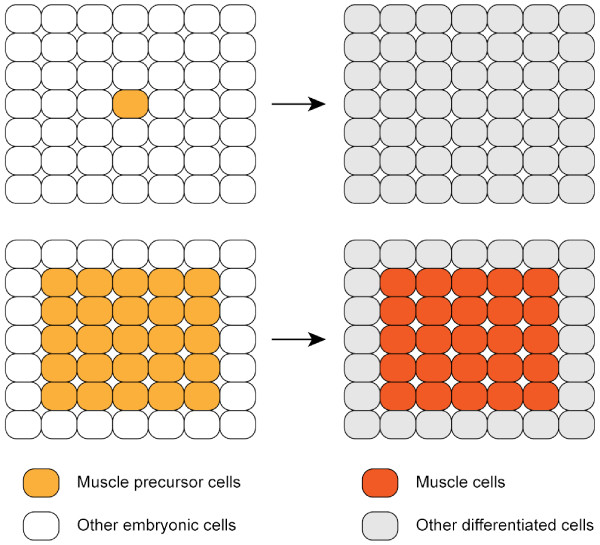
**Diagram depicting the community effect in development**. This figure illustrates the concept of the community effect in an abstract manner. See Introduction for a description of the community effect in muscle development.

The precursor cells generated by an embryonic induction tend to stay together and form a cell group of like character. Despite the fact that those cells often proliferate and their surrounding environment changes as a consequence of morphogenesis, cells in such a group behave as a collective and express the same set of genes that are unique to their differentiation process. One of the mechanisms that control such collective behaviour of cells during animal development is the so-called community effect [1]. A community effect was first discovered in the muscle precursor cells of *Xenopus *embryos [2]. Muscle cells are formed from mesoderm, which itself is generated by an inductive interaction of cells in the equatorial region of blastula embryos in *Xenopus*. Naïve ectoderm cells from blastula embryos change their fate to mesodermal one when juxtaposed to the endodermal tissue that produces the mesoderm-inducing signalling molecules Activin and Xnr (*Xenopus *nodal-related) proteins. Mesoderm cells that contain muscle precursor cells induced in this way or isolated from early embryos can differentiate into muscle cells when cultured as a group of many cells but not as single cells (Figure 1) [3]. This community effect of many nearby muscle precursor cells requires cell-cell interaction mediated by FGF4 (Fibroblast Growth Factor 4) protein. FGF4 (also known as embryonic FGF or eFGF in *Xenopus*) is distinct from the mesoderm-inducing signals [4]. FGF4 and the early mesodermal transcription factor Xbra (*Xenopus *Brachyury) induce expression of each other, thus forming a positive feedback among a group of cells [5,6]. If FGF signalling is blocked by the expression of a dominant negative form of FGF receptor, Xbra expression will be lost [7]. Although it is intuitively apparent that cell-cell communication by diffusible factors plays a crucial role in the community effect, its mechanism is not immediately obvious. Bolouri and Davidson proposed a model of the community effect in sea urchin embryos, which is based on the gene regulatory network operating in the oral ectoderm [8]. In their model, cell-cell communication also plays the central role for the community effect, which is mediated by the *Nodal *gene product. The basic regulatory unit for a community effect thus seems to be a self-activating feedback loop of a gene that expresses extracellular signalling ligands.

The model of Bolouri and Davidson also incorporated an interlinked loop of negative feedback by the Nodal antagonist Lefty. This negative feedback is responsible for restricting the area of Nodal expression within the boundary of the oral ectoderm [9]. Although their model provides an underlying logic to the gene regulatory network of a community effect, many questions still remain unanswered: Is positive feedback signalling among cells sufficient for the community effect? Why does the community effect require many cells? How is such a population size determined? We have addressed these questions theoretically using a combination of stochastic and deterministic modelling methods. We found that a simple linear gene cascade that produces a diffusible factor for cell-cell communication is sufficient to reproduce the community effect in development. We derived the formula for the minimal number of cells required for a community effect and discuss its wider implications for the mechanism of collective behaviour of cells.

## Results

### A minimal model of the community effect in animal development

**Figure 2:**
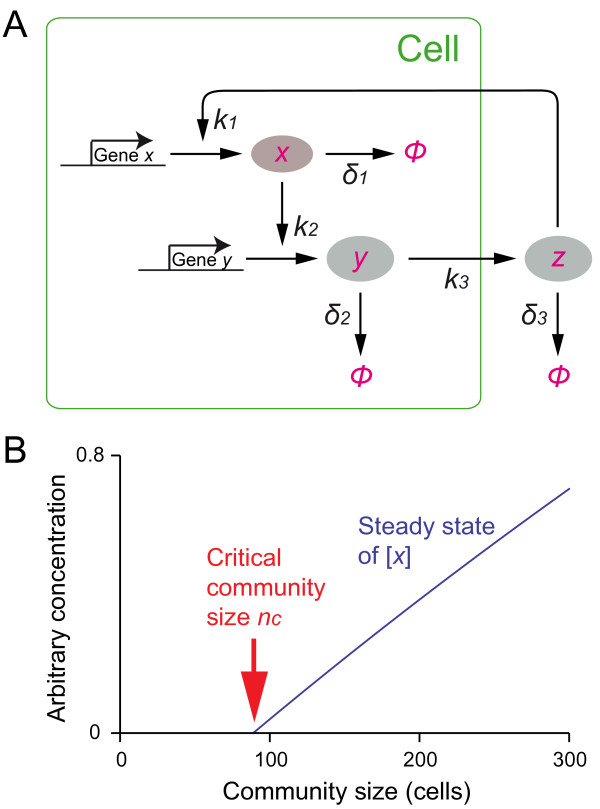
**A minimal model of a community effect**. (A) A schematic depiction of the model. Each molecule or state is indicated in red, and arrows indicate reactions/transitions between those states with reaction rate parameters as indicated. See text for details. (B) Steady state of [*x*] plotted as a function of community size *n*. Parameter values used for the plot are: *k*_1_, *k*_2_, *k*_3 _= 0.02; *δ*_1_, *δ *_2_, *δ *_3 _= 0.01; *V_c _*= 1; *V_s _*= 800.

Our first model of a community effect is based on a simplified abstract scheme as illustrated in Figure 2A. This model does not include transcription (mRNA) steps, and is described by Michaelis-Menten rate equations without Hill coefficient (cooperativity). The system has *n *cells and three components (proteins), *x_i_*, *y_i _*(*i *= 1, 2, ..., *n*) and *z*. *y_i _*is exported from the cell, and is added to the extracellular pool *z*. *z *in turn activates the synthesis of *x_i_*. *z *diffuses into and out of the cell freely. This model explicitly takes account of the system volume *V_s _*(extracellular volume plus total cell volume) and the volume of a cell *V_c_*, both of which remain constant in this model. Note that *V_s _*>*n V_c_*. In the deterministic regime, each cell has identical dynamics. The system is described by a set of ordinary differential equations (ODEs) as follows:(1)

Note that *V_s _*- *n V_c _*is the extracellular volume of the system, so *V_c _*/(*V_s _*- *n V_c_*) is the factor of concentration adjustment. *k*_1_, *k*_2 _and *k*_3 _are the rate constants for production of *x_i_*, *y_i _*and *z*, respectively, and *δ*_1_, *δ *_2 _and *δ *_3 _for degradation. Because cells are identical, Eqs.1 reduce to:(2)

where(3)

The initial condition is *z*_*t *= 0 _> 0 (or *x*_*t *= 0 _> 0 or *y*_*t *= 0 _> 0).

### The condition for a community effect

The ODEs of Eqs.2 can be solved analytically (see additional file 1). We found that above a certain critical cell number *n_c_*, the system is activated and becomes self-sustaining. Figure 2B shows a typical profile of cell activity (concentration of *x*) at the steady state plotted as a function of community size *n*. The *critical community size n_c _*is(4)

where(5)

From Eq.3 and cell density *η *= *n V_c _*/*V_s_*, the *critical cell density *for a community effect *η_c _*is(6)

Note that *ξ*'s numerator is the product of the synthesis rates and the denominator is the product of the degradation rates. Therefore *n_c _*is partly determined by the balance of synthesis and degradation of the components in the positive feedback network. Interestingly, we will reach a similar conclusion about the determinant of *n_c _*for a more elaborate model described below.

### A model of the community effect with transcription

The second model includes transcription (mRNA) steps and is basically a linear combination of the three-stage model of gene expression described in additional file 1, coupled with cell-cell communication. Similar models of gene expression have been adopted in a number of previous studies ([10] for example; see [11] for review).

The model consists of a linear cascade of two genes, *A *and *B*. Gene *A *corresponds to the transcription factor *Xbra *gene and gene *B *is analogous to *FGF4 *gene in *Xenopus*, which is the direct target of Xbra [6]. Protein molecules synthesized from active gene B (*Bp_in_*) are transported out of the cell at the rate *κ*. After diffusing away from the cell that produced them, a secreted extracellular *Bp *(*Bp_out_*) molecule binds to one of the cells in the community irreversibly, and the protein is then converted into another transcriptional activator (*Cp*). This process corresponds in real *Xenopus *embryonic cells to the binding of FGF4 proteins to FGF receptors that activate the signal transduction mediated by the MAP kinase cascade, which in turn induces Xbra expression [12-15].

In the model, the diffusion of *Bp_out_*, its binding to a cell surface receptor and conversion into *Cp *are treated as a single process, which is represented by a combined rate constant *ε*. In this lumped-up process, diffusion of *Bp_out _*and its binding to the receptor is the rate-limiting step because the intracellular FGF signalling mediated by the MAP kinase pathway is thought to be fast relative to those processes. Unless taken up by the cells, *Bp_out _*decays with the rate *δ_d_*, either by degradation or by diffusion away from the cells. *Cp *activates the gene *A*, thus closing the positive feedback loop. A crucial aspect of the model is that this positive feedback is not a simple loop but a complex network with many cells. Also important is that the model has no explicit intracellular feedback mechanism.

For the sake of simplicity of the model, we have made some assumptions. First, a *Bp_out _*protein binds to any cell in the system with equal probability. This assumption is valid if extracellular concentration of *Bp_out _*is similar across the cell community. We have examined whether this is the case in embryos theoretically. In zebrafish embryos, a half-life of FGF8, a member of FGF protein family, was measured to be around 18 min [16] and its diffusion coefficient about 91 *μm*^2^/*s*. We would expect FGF4 proteins in *Xenopus *embryos has similar degradation and diffusion rates and applied them to a simple model of diffusion. From this analysis, we have concluded that the assumption is valid under a certain condition (see additional file 1).

Second, cell division is not considered in the model and cell number remains constant. Third, cells are in a closed system whose size (i.e., volume) is also constant. In real *Xenopus *embryos, the cell community is surrounded by other tissues or cells. Finally, we set the initial condition as follows: at time = 0, both genes *A *and *B *are inactive. In early *Xenopus *embryos, Activin/Xnr signalling induces naïve ectodermal cells to become muscle precursors cells. Instead of introducing another molecule as an inducer into our model, *Bp_out _*substitutes the role and 500 molecules of it is present at time = 0, which rapidly decays (*t*_*1*/2 _≈ 33*min *≪ duration of simulations). This substitution does not affect our analysis because the steady state is independent of the initial condition (see below).

### The deterministic model and the condition for a community effect

The model we have just described is represented by a set of rate equations:(7)(8)(9)(10)(11)(12)(13)(14)

**Figure 3:**
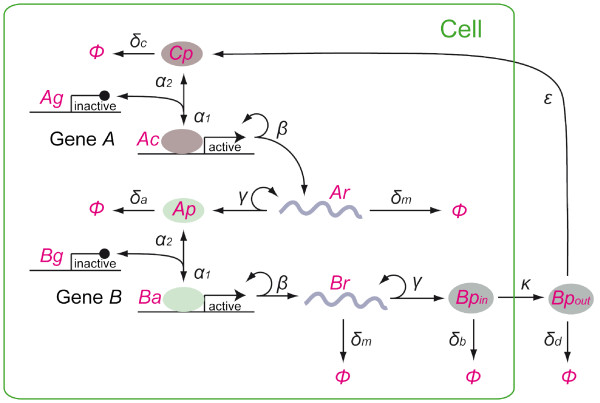
**A model for the community effect in development**. See main text for details. Each molecule or state is indicated in red, and arrows indicate reactions/transitions between those states with reaction rate parameters as indicated.

Here, [*Ag*] + [*Ac*] = [*Bg*] + [*Ba*] = 1 because of the conservation of the gene copy numbers. In Eq.13, *ε *is multiplied by *n *because each cell acts as a sink for *Bp_out_*. All variables in Eqs.7-14 are the number of each molecule (per unit volume) per cell except [*Bp_out_*], which represents the number of extracellular *Bp*. All other reaction rates are depicted by the arrows in Figure 3.

**Figure 4:**
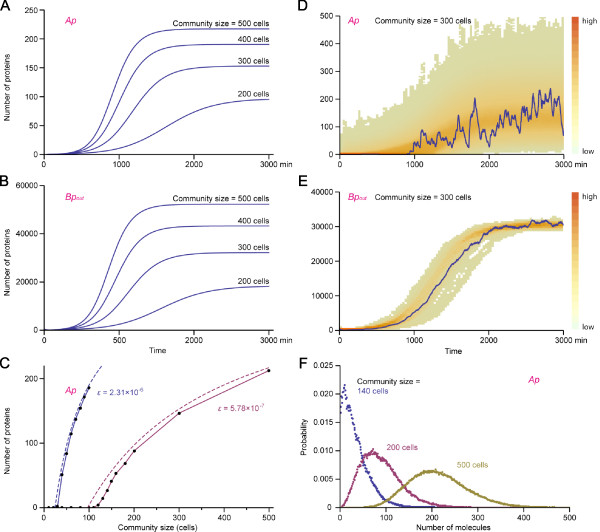
**Simulation results of the community effect model**. (A, B) Numerical simulations of the deterministic rate equations Eqs.7-14. (A) is the plot for [*Ap*] and (B) for [*Bp_out_*]. Simulation results for different community size are shown. With 100 cells, very little gene expression occurs at steady state (not shown) as 100 cells are close to *n_c _*≈ 97. (C) Average number of *Ap *at steady state (10000 min) as a function of community size (solid lines). Dotted curves indicate number of *Ap *at steady state ([*Ap*]*) obtained by Eqs.35 in additional file 1. Plots are shown for *ε *= 2.31 × 10^-6 ^and 5.78 × 10^-7^. (D, E) Time series of [*Ap*] and [*Bp_out_*] as overlays of 100 stochastic simulation results (temperature map) for community size of 300 cells and *ε *= 5.78 × 10 ^-7^. Solid lines show a typical simulation result. (F) Probability distributions of [*Ap*] at steady state (*t *= 10000 min in stochastic simulations) for community size *n *= 140, 200, and 500 cells. *ε *= 5.78 × 10 ^-7^. All simulations in this figure are with one gene copy each for gene *A *and gene *B*.

We first performed numerical simulations of the deterministic rate equations Eqs.7-14 (parameter values in Table 1). Figure 4A and 4B show the time course of [*Ap*] and [*Bp_out_*] (*ε *= 5.78 × 10^-7^). The analysis revealed that following induction at *t *= 0 with [*Bp_out_*] = 500, gene expressions increase after a time lag and become self-sustaining. But the cell's activity becomes self-sustaining only when the number of cells present is above a certain critical number *n_c_*. This is a community effect, which is analogous to the one observed in *Xenopus *embryos. We asked how this critical community size *n_c _*is determined. In fact, *n_c _*can be derived from the steady state solution of Eqs.7-14 as(15)

**Table 1:** Parameter values used in simulations

Parameter	Reaction	**Parameter value **(**sec ^-1^**)	**t**_**0**.**5**_**(approx.)**
*α*_1_	Binding of transcriptional activator to promoter	1.93 × 10 ^-4^	1 hr

*α *_2_	Dissociation of transcriptional activator from promoter	3.47 × 10 ^-2^	20 sec

*β*	Transcription	1.16 × 10 ^-2^	1 min

*γ*	Translation	2.31 × 10 ^-2^	30 sec

*δ_a_, δ_b_, δ_c_, δ_d_*	Degradation/disappearance of proteins	3.47 × 10 ^-4^	33 min

*δ_m_*	Degradation of mRNA	1.16 × 10 ^-3^	10 min

*κ*	Exocytosis	3.85 × 10 ^-4^	30 min

ε	'Communication' by extracellular factor	2.31 × 10 ^-6 ^or 5.78 × 10 ^-7^	83 or 333 hrs

Simulation parameters are all within biologically or biochemically relevant range, which usually spans orders of magnitude, such as the one for protein degradation rates. Where possible, we have referred to reaction rates that have been determined experimentally to choose parameter values for simulations (for example, [39-45]). *ε *is an abstract parameter and its value for stochastic simulations was estimated and chosen so that a community effect could be realised *in silico*.

where(16)

(see additional file 1). *ρ *must be larger than 1 in order to have a community effect for any community size. With the parameter values used in the simulations (Table 1), we obtain *ρ *≈ 7.2 and the critical community size *n_c _*≈ 97. Comparing the diagram of the model (Figure 3) and Eq.16, the meaning of *ρ *becomes apparent: the numerator of *ρ *is the product of the rate constants of all reaction steps that promote the cascade of gene expressions in the cell, while the denominator is the product of the rates of reactions in the opposite direction such as protein degradation. An important conclusion drawn here is that *n_c _*is independent of the initial condition. Therefore, so long as [*Bp_out_*] > 0 at *t *= 0, a community effect will be triggered when the community size *n *>*n_c_*, although in stochastic simulations [*Bp_out_*] at *t *= 0 must be sufficiently large (see below). We have already seen a similar condition for a community effect with a simplified model of the community effect that omits transcription steps (for which *n_c _*is described in Eqs.4 and 5, see Figure 2). When *ξ *≫ 1, *n_c _*is proportional to 1/*ξ*. Similarly from Eq.15, when *ρ *≫ 1, *n_c _*is proportional to 1/*ρ*.

### Gene copy number and the critical population size for a community effect

We next asked how copy number of genes affects the community effect in our model. This is an important question to ask because certain types of genetic disorders such as cancer may be the consequence of a mutation in one of the two copies of the gene that is required for a community effect (see Discussion). When multiple copies of genes are present per cell, such as diploid cells, the critical cell number *n_c _*for a community effect is:(17)

where *a *and *b *are the copy numbers of gene *A *and gene *B*, respectively and *ρ *is defined in Eq.16 (see additional file 1, Eq.38 for derivation). The critical community sizes *n_c _*are 22 for *a *= 2, *b *= 2, and 45 for *a *= 1, *b *= 2 according to Eq.17 with the parameters listed in Table 1 and *ε *= 5.78 ×10^-7^. These values are in good agreement with the stochastic simulation results (see below). Therefore, *n_c _*for the community of heterozygous diploid cells, i.e., one copy of gene *A *is defective, is larger than that of the community of homozygous diploid cells. Gene copy number also significantly affects the expression of the genes at steady state (see below).

### Stochastic simulations of the community effect model

We next performed stochastic simulations over a range of *n *communicating cells. Because a community effect concerns the heterogeneity of a cell population, noise in gene expression becomes an important aspect that needs to be examined by stochastic simulations. We used the same parameter values as those for the three-stage model of gene expression (Table 1). Figure 4D and 4E show a plot of [*Ap*] and [*Bp_out_*] over time in the simulations with *n *= 300. After an almost quiescent time lag, [*Ap*] and [*Bp_out_*] increase and reach steady distributions, and their expressions become self-sustaining. The steady-state average of [*Ap*] in the stochastic simulations (solid lines in Figure 4C) is in good agreement with that of the deterministic rate equations (dotted lines in Figure 4C, which are calculated by Eqs.35 in additional file 1).

**Figure 5:**
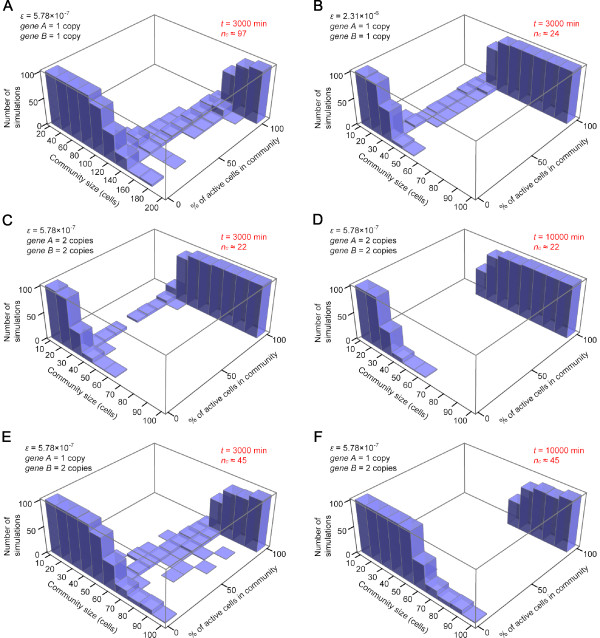
**Community effect observed in stochastic simulations**. Distributions of percentage of active cells in the community for a range of community size as indicated. 100 simulations were performed for each community size. Percentage of active cells ([*Ap*]> 0) at the end of simulation was calculated for each simulation, plotted as a histogram, which are combined as 3D plots. (A) The histogram for *ε *= 5.78 × 10 ^-7^, *a *(copy number of gene *A*) = 1, *b *(copy number of gene *B*) = 1. (B) *ε *= 2.31 × 10 ^-6^, *a *= 1, *b *= 1. (C, D) *ε *= 5.78 × 10 ^-7^, *a *= 2, *b *= 2. (E, F) *ε *= 5.78 × 10 ^-7^, *a *= 1, *b *= 2. (A, B, C, E) are the histograms at *t *= 3000 min in the simulations and (D, F) at t = 10000 min. Note that (C) and (D) are obtained at different time points from the same set of simulations, so are (E) and (F). Histograms for *a *= 2, *b *= 1 are similar to Fig. 5E and F (data not shown).

We performed 100 simulations for each community size *n*, and calculated the percentage of active cells ([*Ap*] > 0) at the end of each simulation: the results are shown for haploid (Figure 5A, B), homozygous diploid (C, D) and heterozygous diploid cell communities (E, F; also see the next section). When the community size is below the critical cell number *n_c _*(indicated in each panel in red), the community effect does not occur and all cells become quiescent. In contrast, when the community size *n *is larger than *n_c _*but close to it (*n *≈ *n_c_*), the behaviour of cells becomes unpredictable: sometime all the cells are active while in other occasions they are all inactive or only partially active (Figure 5A, B, C and 5E, *t *= 3000 min). However, this heterogeneity is transient and the community eventually become homogeneous after a sufficiently extended time period (Figure 5D and 5F, *t *= 10000 min). These results indicate the probabilistic nature of a community effect when *n ≈ n_c _*, that is, the system can end up with either of two stable states with a finite probability. In contrast, if the community size is sufficiently large (*n *≫ *n_c_*), all cells become active (Figure 4F and Figure 5).

### Gene expression control by a community effect

During embryogenesis, cell differentiation usually accompanies cell proliferation. Before terminal differentiation, precursor cells must divide and achieve a certain population size. But at the same time they must maintain a constant gene expression profile that is required for them to become specific tissue such as muscle. We asked how the amount of gene expressions changes if community size grows in our scenario of a community effect.

**Figure 6:**
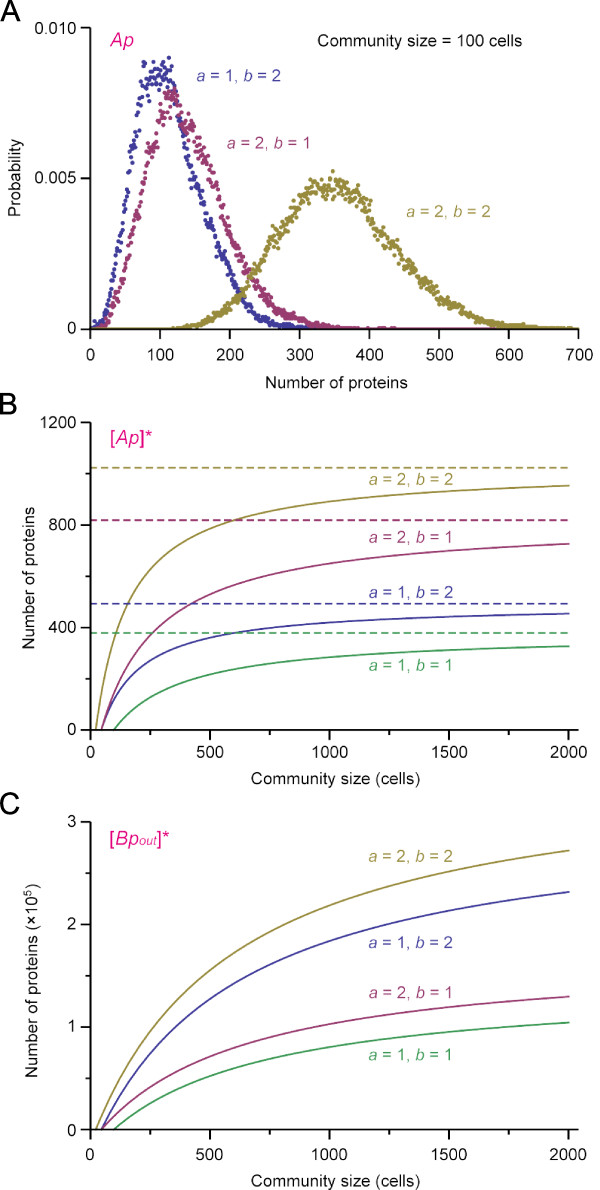
**Influence of gene copy number on gene expressions at steady state**. (A) Probability distribution of [*Ap*] at the end of stochastic simulations for the community size *n *= 100 (*t *= 10000 min). Plots for different combinations of gene copy numbers are shown as indicated. (B) [*Ap*] at steady state ([*Ap*]*) is plotted as a function of community size for different gene copy numbers as indicated. [*Ap*]* is calculated according to Eqs.35 in additional file 1 with parameter values in Table 1, *ε *= 5.78 × 10 ^-7^. Dotted lines are the theoretical maxima . (C) [*Bp_out_*] at steady state ([*Bp_out_*]*) is plotted as a function of community size for different gene copy numbers. Parameter values are the same as in (B). [*Bp_out_*]* also approaches to the theoretical upper limit  (not shown;  ≈ 358000 for *a *= 2, *b *= 2; 308000 for *a *= 1, *b *= 2; 172000 for *a *= 2, *b *= 1; 143000 for *a *= 1, *b *= 1).

Figure 6A shows that the heterozygous diploid cell community (*a *= 1, *b *= 2 or *a *= 2, *b *= 1) have a diminished expression of gene *A *(and gene *B*, data not shown) compared to the homozygous diploid cell community (*a *= 2, *b *= 2). Figure 6B shows [*Ap*] at steady state ([*Ap*]*) as a function of community size *n *with different gene copy numbers. The plot indicates that, even when cells in the community continue to proliferate, [*Ap*]* (and also [*Bp_in_*]*, data not shown) of the heterozygous diploid cells never reaches that of normal homozygous cells. The analysis revealed that [*Ap*]* has a theoretical upper limit  (dotted lines in Figure 6B). Therefore, the compromised gene expression in the heterozygous diploid cell community cannot be compensated by increasing its population size.

Figure 6B also indicate that [*Ap*]* remains fairly constant for large cell community. This is advantageous for the community because the gene expression is not affected by the change of cell number. In contrast, the steady state level of *Bp_out _*([*Bp_out_*]*) increases as the community size grows (Figure 6C). [*Bp_out_*]* is also orders of magnitude larger than [*Ap*]* for a large community. Therefore, [*Bp_out_*] works as a collective 'memory' of cells while the activity (gene expressions) of each cell is still weak after the induction of the community.

We next asked how different environments outside the cell affect gene expressions. It is easily imaginable that the extracellular environment changes during embryogenesis. This should alter *ε*, which reflects how fast cells communicate with each other (i.e. the average distance between cells), and *δ_d _*that defines how fast *Bp_out _*decays or drifts away from the cells.

**Figure 7:**
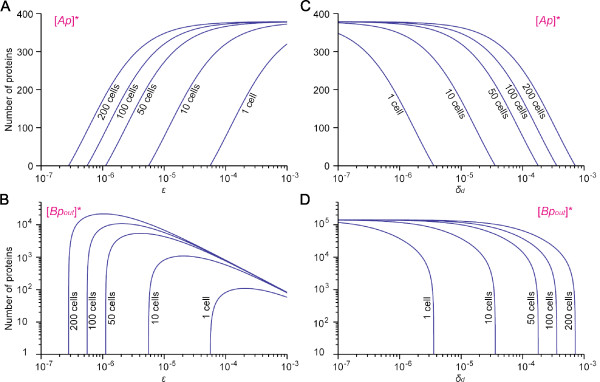
**Influence of the cell-cell communication rate *ε *and the decay rate of extracellular factor *δ_d _*on gene expressions**. Protein number of *Ap *and *Bp_out _*at steady state for different communitysizes are plotted as a function of *ε *(A, B) and *δ_d _*(C, D). These plots are with gene copy numbers *a *= 1, *b *= 1, but qualitatively similar plots can be obtained with different gene copy numbers.

Figure 7 shows steady-state activity [*Ap*]* and [*Bp_out_*]* as a function of *ε *(Figure 7A, B) and *δ_d _*(Figure 7C, D). In Figure 7A and 7B, the intersection of each line for constant cell number with the axis of *ε *corresponds to the value of *ε *for which that cell number is *n_c_*. Similar argument can be applied in Figure 7C and 7D to the value of *δ_d _*with regard to *n_c_*. Figure 7A indicates that for the large population *n *≫ *n_c_*, [*Ap*]* is independent of *ε*. Therefore, a small fluctuation of has little influence on the expression of *Ap *at steady state. In contrast, [*Bp_out_*]* can change dramatically in response to a slight change in *ε *if *n ≈ n_c_*. For example, for *ε *= 1.12 × 10 ^-6 ^and *n *= 50 (≈ *n_c_*), [*Bp_out_*]* is ≈ 23 but increases to ≈ 3200 when *ε *changes upward by half (*ε ≈ *1.68 10 ^-6^) while it becomes 0 when *ε *changes downward by the same amount (*ε ≈ *0.56 × 10 ^-6^). On the other hand, when *n *≫ *n_c_*, [*Bp_out_*]* is relatively insensitive to small changes in *ε*. Similarly, [*Ap*]* and [*Bp_out_*]* is independent of *δ_d _*when *n *≫ *n_c_*. For *n *≫ *n_c_*, a small perturbation of *δ_d _*barely influences [*Bp_out_*]* or [*Ap*]*. In contrast, [*Bp_out_*]* could change drastically when *n *≈ *n_c _*(Figure 7C, D).

We also examined how noise in gene expression is affected by the change in *ε *when *n *≫ *n_c_*, in other words, how robust cell's activity is to changes of extracellular environment. Noise (coefficient of variation) was calculated from the stochastic simulation results described above. Table 2 summarises the calculations. It turned out that, as system size (i.e., tissue size) grows without cell proliferation (i.e., with smaller *ε*), the noise of the steady state gene expression [*Ap*]* becomes larger. On the other hand, as cell number in the community grows with a constant system size, gene expression noise decreases. Therefore, when cell number and system size increase at the same time, the effect of these cancels out and noise in gene expression would remain at the same level.

**Table 2:** Noise in gene expression depends on community size and communication rate *ε*

Community size (cell number)	*ε *(**sec ^-1^**)	Mean	Standard deviation	Noise
200	2.31 × 10 ^-6^	260.8	70.0	0.268

200	5.78 × 10 ^-7^	87.7	43.1	0.492

300	5.78 × 10 ^-7^	146.6	54.7	0.373

500	5.78 × 10 ^-7^	212.7	63.7	0.300

Noise (coefficient of variation = standard deviation/mean) in gene expression was calculated from the steady state distribution of [*Ap*]*, which was derived from the stochastic simulations with one copy each of genes *A *and *B*, and with parameter values as listed in Table 1.

The above observations indicate that if a cell community is dispersed (i.e., *ε *→ 0 and *δ_d _*→ ∞), cells cannot keep the self-sustaining gene expressions of a community effect. Conversely, the gene expressions are maintained as long as cells are close enough to each other (*ε *above the threshold) and the community is insulated to prevent too much loss of *Bp_out _*(*δ_d _*below the threshold). These are the hallmarks of a community effect.

## Discussion

### The mechanism of the community effect in development

The pioneering experimental work by Gurdon et al. [2-4] and the recent theoretical work by Bolouri and Davidson [8] have suggested that the feedback cycle of cell-cell communication by diffusible signalling proteins and their self-induction is essential for a community effect. However, it has been unknown whether this positive feedback among cells by cell-cell communication is sufficient or an additional layer of interlinked gene regulation is necessary for a community effect. Nor has it been clear how the size of a cell community to bring about a community effect is determined. Our present work has provided a theoretical basis for the community effect and demonstrated the crucial role of the positive feedback between cells by diffusible factors. Our model with a simple linear gene cascade and cell-cell communication by signalling factors reproduces the main characteristics of a community effect, that is, (1) it requires many cells, (2) cells in the community must be near each other, and (3) the gene expressions for the community effect becomes self-sustaining after the initial transient induction.

With regard to the condition for a community effect, the same conclusion has been drawn from the simplified minimal model and the more elaborate model with transcription steps. The analytical formula for the critical cell number of the community *n_c _*(Eqs.4 and 15) explains the above characteristics (1) and (2) of the community effect: first, *n_c _*is determined by the balance of synthesis and degradation of the components involved, i.e., mRNAs and proteins; second, it is governed by the parameter that reflects how fast cells can communicate with each other (*k*_1 _or *ε*), and also by how close cells are located to each other. In other words, the cell density of the community must be above a critical threshold to have a community effect. This is the most fundamental principle of the community effect, which has been observed experimentally but its theoretical basis has been unknown. When the size of the community is less than *n_c_*, there is only one steady state with all cells being quiescent.

### The intrinsic property of the cell community dictates the community effect

An important insight from our model analysis is that the expression level of genes at self-sustaining steady state ([*Ap*]* and [*Bp_in_*]*) is independent of the magnitude of the initial induction. The analytical solution of the steady state (Eqs.35 in additional file 1) indeed contains no term for the initial condition. But the system's dynamics changes according to the initial transient induction, i.e., the higher, or the longer the initial induction, the shorter the time required for the system to reach the steady state (data not shown). Therefore, it is the intrinsic property such as the community size that dictates the community effect and the outcome (i.e., steady state gene expressions). It is not the extrinsic inductive signal that is required for a community effect *per se*, although it is required to trigger the community initially. Our model analysis also suggests that this intrinsic self-organising property of the community requires no explicit intracellular feedback mechanism, but only a linear cascade of a signal transduction and its downstream gene expressions of signalling molecules for cell-cell communication.

Nevertheless, a community of precursor cells must receive sufficient inductive signals because the lag period before steady state (Figure 4A, B, D and 4E) must be within the time frame of the program of embryogenesis [17]. And also the amount and duration of the inductive signal, which acts as a morphogen in a concentration-dependent manner [18], must be within the right range. For instance in early *Xenopus *embryos, naïve ectodermal cells must receive a high concentration of Activin/Xnr signal to become muscle precursor cells. For the patterning of ventral neural tube by the morphogen Sonic Hedgehog (Shh) in vertebrates, the duration of Shh signalling is critical for the morphogen interpretation [19]. In our model, the length of the initial induction also affects the system's dynamics as mentioned above. With an adequate level and duration of the initial induction, the community effect would be fully activated at the right time and the precursor cells can proceed to the next step of differentiation.

### The community effect and pattern formation in development

Although our model can reproduce the qualitative hallmarks of the community effect quite well, it cannot provide the mechanism that restricts the area of the community effect within a boundary. Simulations of our model indeed showed that the community effect spreads across the whole community (data not shown), indicating that an additional control mechanism is necessary to restrict the community effect within the boundary of an embryonic field. Such mechanism must be essential for patterning the embryo. One obvious candidate for that is a negative feedback regulation by an antagonist of the diffusible factor for cell-cell communication, as indicated in the model of Bolouri and Davidson [8], although such additional control is dispensable for the community effect.

For the sake of simplicity, we have omitted the spatial information and presumed that the cells in a community are well mixed in our model. Therefore it remains unknown how the spatial arrangement of the cells influences the community effect. This question is highly relevant to the mechanism of morphogenesis and the patterning of embryonic tissues. It may well be the case that the incorporation of explicit spatial information (i.e., the cells' relative position to each other in the community) and diffusion term of the extracellular factors partly solves the problem of unrestricted spread of the community effect in the current model (also see additional file 1). The importance of the spatial distribution of cells has also been highlighted for quorum sensing of bacteria [20], a process that is similar to the community effect (see below). It remains to be seen how the community effect is coupled with the mechanism of pattern formation in development.

### Robust gene expression control by a community effect

We have found that the community effect is a robust control mechanism to keep uniform gene expressions across a group of cells. When the community size is sufficiently large (*n *≫ *n_c_*), gene expressions for the community effect become independent of *ε*, which reflects the average distance between each pair of cells in the community, and *δ_d_*, which defines how fast *Bp_out _*decays or diffuses away form the community (Figure 7). In other words, a small fluctuation of the extracellular environment has little influence on the gene expression of a large uniform cell community. This is an advantage for the cells undergoing morphogenesis, because that allows cells to move as long as they are close to each other.

We also found that, although an individual cell's activity is weak during the lag period just after the initial induction (Figure 4), the pool of extracellular protein (*Bp_out_*) accumulates quickly and becomes large enough to buffer the fluctuations of gene expressions of each cell. The community effect is thus a simple yet robust mechanism to keep the uniform collective behaviour of cells, especially in the changing environment during embryogenesis.

### Community effect as a mechanism of size control and tumorigenesis

How size is controlled in embryos and in adults remains an intriguing problem in biology. Size control is linked to pattern formation during embryogenesis, which can be viewed as the partitioning of the limited mass of an embryo. Processes of cell proliferation, growth (increase of cell mass) and death are all part of the size control and their balance determines the size of tissues in embryos, and ultimately that of the organs in adult bodies [21]. But how do tissues or organs sense their size and execute those processes? A community effect may be a strong candidate for that mechanism because it arises from the intrinsic self-organising property of the cell population as our theoretical work suggests.

As a mechanism of the size control, a community effect stops cell proliferation and growth, and promotes cell death when the cell population and its density in the tissue or organ exceeds a certain threshold. This regulation can be achieved by placing a given component in the gene circuit of the community effect (e.g., *Ap *in our model) upstream of the cell cycle/cell death regulators or growth factors. This sort of system allows tissues and organs to limit their size autonomously. The community effect could be part of the mechanism not only of size control but also of tissue homeostasis in general. However, the mechanism of this sort is vulnerable when something goes wrong with the community effect. The positive feedback mechanism is indeed implicated in the onset of autosomal dominant diseases [22,23].

Let us consider diploid organisms for example. If one of the two copies of gene *A *in our model has become defective due to a mutation of the gene, its expression at steady state [*Ap*]* is greatly compromised (Figure 6A). If the compromised steady state expression is below the threshold for size control, cells in the tissue or organ continue to proliferate and grow indefinitely in theory because the loss of gene *A*'s expression cannot be compensated by increasing the number of cells in the community, as we have seen in our model analysis. This may explain the origin of a certain type of cancer. Recent studies have demonstrated that haploinsufficiency of tumour suppressor genes contributes to tumorigenesis (reviewed in [24]). In fact, it has been proposed that disruption of a quorum sensing mechanism triggers tumorigenesis [25]. Conversely, the abnormal amplification of gene *A *or gene *B *in our model may lead to the premature termination of a tissue growth, or atrophy.

### Similarity between the community effect in development and the quorum sensing in bacteria

The community effect in development is a typical example of a collective behaviour of cells, which seems to be quite universal and can be found not only in metazoans but also in microorganisms, for instance, the quorum sensing of bacteria. Although there is a number of different mathematical models of quorum sensing, all known quorum-sensing systems have the same network architecture (reviewed in [26]): first, low molecular-weight molecules called autoinducers are synthesized and released by the cells; second, these autoinducers bind to cognate receptors in the cells, which in turn induce their own production as well as the enzyme that catalyse the production of autoinducers. This leads to change in gene expressions across the cell population. The fundamental architecture of quorum-sensing network is therefore analogous to that of community effect and the positive feedback of cell-cell communication lies at the heart of these two disparate systems of collective cellular behaviour. The same principle for a community effect described in this work may thus apply to quorum sensing as well. To our knowledge, this mechanistic similarity between the community effect and quorum sensing has never been discussed. The quorum sensing network structure is, however, different from that of the community effect and has a pair of interlinked positive feedbacks. This additional complexity is responsible for the switch-like behaviour of the network [27], which may enhance the system's population-dependent response (i.e., community effect).

Chen and Weiss constructed an artificial quorum sensing system in yeast S.cerevisiae [28]. They integrated the *Arabidopsis *cytokinin production and its receptor components with the cell's endogenous osmotic stress sensing signalling pathway. This synthetic hybrid circuitry allows cells to communicate with each other and confers quorum sensing. Incidentally, their artificial quorum-sensing system has demonstrated experimentally that a simple signal transduction that stimulates the production of a diffusible factor for cell-cell communication is sufficient for a collective behaviour of cells, i.e., quorum sensing. This is consistent with our theoretical analysis of the community effect model, which has similar network architecture.

For both community effect and quorum sensing, cell density must be above the critical threshold. Our theoretical analysis has also indicated that cells need to be in an insulated system to minimize the loss of extracellular signalling factors from the community, especially just after the induction before the system reaches fully-activated steady state. Conversely, these principles may be relevant to collective behaviour of cells in general and prove to be a useful guidance to tissue engineering and biotechnology. For example, they may be applied to maintain stem cells and direct their differentiation in vitro, or to engineer bacterial cells that activate gene expressions when the cell population reaches a critical cell density [29].

## Conclusions

Our model analysis indicates that a linear gene cascade with positive cell-cell interactions is sufficient to reproduce the community effect in animal development. The critical community size required for a community effect is determined by the balance of synthesis and degradation of the components involved in the process, as well as the cell density. It suggests that the community's long-term behaviour is independent of the initial induction level, although the initiation of a community effect requires a sufficient amount of inducing signal. The mechanism of the community effect is analogous to that of quorum sensing in bacteria. The community effect may underlie the size control in animal development and also the genesis of autosomal dominant diseases including tumorigenesis.

## Methods

### Stochastic simulations

Stochastic simulations were performed using the well-established Gillespie Monte Carlo algorithm [30], which is based on the theorem of Joseph L. Doob, one of the founders of theory of stochastic processes [31]. The algorithm offers an exact solution to the stochastic evolution of a system of chemical reactions. We used two different platforms that make use of the algorithm, SPiM (the Stochastic Pi-Machine [32]) and a tailored stochastic C-code. SPiM is a simulator package based on the stochastic Pi-calculus [33], which has been applied recently to modelling of chemical reactions and biological systems [34,35]. The codes used in this study are provided in additional file 2 and 3 (stochastic Pi-calculus) and additional file 4 (C code). Detailed description of SPiM including how to run simulations is available at the web site [32]. The SPiM codes used in this study have been built upon the earlier works [36-38].

### Mathematical analysis of the model

Mathematica^® ^(Wolfram Research) was used for the mathematical analysis and numerical integration of the deterministic models, and also for statistical analysis of stochastic simulations.

## Authors' contributions

YS conceived of the study, designed it, performed the model analysis and simulations by the stochastic pi-calculus, and drafted the manuscript. CL and CK participated in the design of the study and the model analysis, performed simulations by the stochastic pi-calculus, and helped to draft the manuscript. EU participated in the model analysis, performed simulations by the C codes and helped to draft the manuscript. MT participated in the model analysis and helped to draft the manuscript. All authors read and approved the final manuscript.

## Supplementary Material

Additional file 1 **The deterministic community effect models, derivation of the critical community size and a model of diffusion in a spherical tissue**. Details of the mathematical analysis is described in this file for (1) a minimal model of a community effect (Eq.2), (2) a three-stage model of gene expression and (3) the complete model with transcription (Eqs.7-14) and (4) a model of diffusion in a spherical tissue. Click here for file

Additional file 2 **SPiM code for the three-step gene expression**. The file is in simple text format (.txt) and runs in SPiM without changing its format. The name of the file may need to be changed without spaces, e.g., AdditionalFile2.txt in order to run simulations by the command line version of SPiM. Click here for file

Additional file 3 **SPiM code for the community effect model**. This file is also in simple text format (.txt) and runs in SPiM without changing its format. The name of the file may need to be changed without spaces, e.g., AdditionalFile3.txt to run simulations. This SPiM code is for 10 cells with two copies each of gene *A *and gene *B*. You would not see any interesting simulation results with 10 cells, but need to increase the community size to at least above 22 cells to have a community effect with the parameter values in the code. Click here for file

Additional file 4 **C code for the community effect model**. This C-program was written by EU. In order to run the program, it requires the random number generators (C codes called <gasdev.c>, <nrutil.c> and <ran1.c>) provided in [46] and a C-compiler installed on your computer. It can be opened and read by a standard text editor. Click here for file
