# Supplementary material for: Theoretical basis of the community effect in development
Source: BMC Syst Biol. 2011 Apr 17;5:54. doi: 10.1186/1752-0509-5-54 (PMC3105943; doi:10.1186/1752-0509-5-54)
Supplement: Additional file 1 — The deterministic community effect models, derivation of the critical community size and a model of diffusion in a spherical tissue. Details of the mathematical analysis is described in this file for (1) a minimal model of a community effect (Eq.2), (2) a three-stage model of gene expression and (3) the complete model with transcription (Eqs.7-14) and (4) a model of diffusion in a spherical tissue. [file 1752-0509-5-54-S1.PDF]

Theoretical basis of the community effect in development

Yasushi Saka, Cédric Lhoussaine, Céline Kuttler, Ekkehard Ullner and Marco Thiel

Additional File 1

Details of the mathematical analysis is described in this file for (1) a minimal model of a community effect (Eqs.2 in the main text), (2) a three-stage model of gene expression, (3) the complete model with transcription (Eqs.7~14 in the main text) and (4) a model of diffusion in a spherical tissue.

February 2, 2011

A minimal model of a community effect

■ The condition for a community effect: linear stability analysis

The fixed points of Eqs.2 in the main text are

$$\{x^* = 0,\ y^* = 0,\ z^* = 0\} \tag{18}$$

and

$$\left\{x^* = \frac{k_1 k_2 \mu - \delta_1 \delta_2 \delta_3}{\delta_1 (k_2 \mu + k_2 \delta_3 + \delta_2 \delta_3)},\ y^* = \frac{k_1 k_2 \mu - \delta_1 \delta_2 \delta_3}{\delta_2 (k_1 \mu + \delta_1 \mu + \delta_1 \delta_3)},\ z^* = \frac{k_1 k_2 \mu - \delta_1 \delta_2 \delta_3}{\delta_3 (k_1 k_2 + k_1 \delta_2 + \delta_1 \delta_2)}\right\}. \tag{19}$$

The Jacobian at one of the fixed points $\{x^* = 0,\ y^* = 0,\ z^* = 0\}$ is

$$A_1 = \begin{pmatrix} -\delta_1 & 0 & k_1 \\ k_2 & -\delta_2 & 0 \\ 0 & \mu & -\delta_3 \end{pmatrix}.$$

The characteristic polynomial of A_1 is

$$p_1(\lambda) = k_1 k_2 \mu - (\lambda + \delta_1)(\lambda + \delta_2)(\lambda + \delta_3). \tag{20}$$

Its eigenvalues (roots of characteristic equation $p_1(\lambda) = 0$) are either three reals (two of them can be identical/degenerate), or one real and two complex numbers that are conjugate to each other. λ values at the intersections of

$$\begin{aligned} w &= (\lambda + \delta_1)(\lambda + \delta_2)(\lambda + \delta_3) \\ w &= k_1 k_2 \mu \end{aligned} \tag{21}$$

are the (real) eigenvalues. Note that $k_1 k_2 \mu > 0$. Let $\delta_1 \geq \delta_2 \geq \delta_3 > 0$ and the eigenvalues be one real (λ_1) and two complex numbers (λ_2, λ_3),

$$\begin{aligned} \lambda_1 &= a \\ \lambda_{2,3} &= b \pm c\, i \\ a, b, c &\in \mathbb{R}. \end{aligned} \tag{22}$$

Below is a plot of Eqs.21:

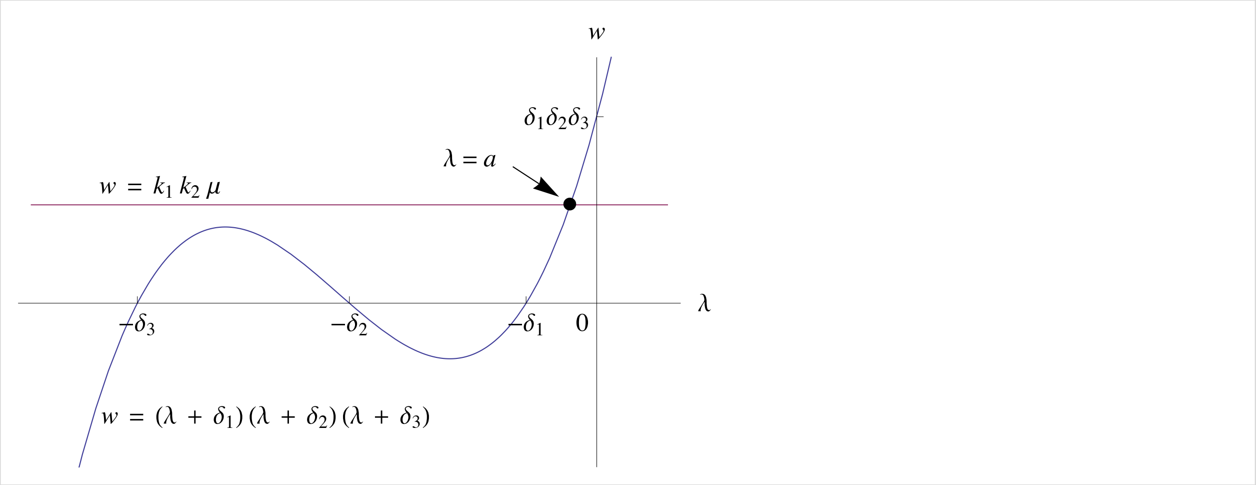

Fig. S1 A plot for the characteristic polynomial.

If $\{x^* = 0,\ y^* = 0,\ z^* = 0\}$ is a stable fixed point, $a < 0$ and $b < 0$. The condition for $a < 0$ is

$$p_1(0) = k_1 k_2 \mu - \delta_1 \delta_2 \delta_3 < 0 \Rightarrow \delta_1 \delta_2 \delta_3 > k_1 k_2 \mu . \tag{23}$$

Because the sum of the eigenvalues equals the sum of diagonals of A_1

$$\lambda_1 + \lambda_2 + \lambda_3 = -(\delta_1 + \delta_2 + \delta_3) = a + 2b \tag{24}$$

and $a > -\delta_1$ (hence $a + \delta_1 > 0$), thus

$$b = -\frac{a + \delta_1 + \delta_2 + \delta_3}{2} < 0. \tag{25}$$

Therefore, Eq.23 is the condition for $a, b < 0$. When the eigenvalues are all reals (i.e., when there are two or three intersections in the plot above), they must be all negative if $\{x^* = 0,\ y^* = 0,\ z^* = 0\}$ is a stable fixed point. The condition for that is also Eq.23.

The Jacobian at the other fixed point (Eqs.19) is

$$A_2 = \begin{pmatrix} -\delta_1 & 0 & -\frac{z k_1}{(1+z)^2} + \frac{k_1}{1+z} \\ -\frac{x k_2}{(1+x)^2} + \frac{k_2}{1+x} & -\delta_2 & 0 \\ 0 & -\frac{y \mu}{(1+y)^2} + \frac{\mu}{1+y} & -\delta_3 \end{pmatrix}$$

and its characteristic polynomial is

$$p_2(\lambda) = \frac{\delta_1^2 \delta_2^2 \delta_3^2}{k_1 k_2 \mu} - (\lambda + \delta_1)(\lambda + \delta_2)(\lambda + \delta_3). \tag{26}$$

Using a similar arguments as above, the condition for the stable fixed point is

$$p_2(0) = \frac{\delta_1^2 \delta_2^2 \delta_3^2}{k_1 k_2 \mu} - \delta_1 \delta_2 \delta_3 < 0 \Rightarrow \delta_1 \delta_2 \delta_3 < k_1 k_2 \mu. \tag{27}$$

This is the reciprocal condition to Eq.23, hence the system described by Eqs.2 has one stable and one unstable fixed point. Their stabilities are exchanged at

$$\delta_1 \delta_2 \delta_3 = k_1 k_2 \mu \tag{28}$$

as the parameter value μ changes (in mathematics jargon, it is called transcritical bifurcation).

■ Critical community size for a community effect in the minimal model

Substituting μ in Eq.28 with Eq.3 and solving for n yields

$$n = \frac{V_s\, \delta_1\, \delta_2\, \delta_3}{V_c\, (k_1\, k_2\, k_3 + \delta_1\, \delta_2\, \delta_3)}. \tag{29}$$

This leads to Eqs.4 and 5 in the main text.

If community size $n > n_c$, the system is activated and becomes self-sustaining, because one of the fixed point (Eqs.19) is stable while the other one (zero fixed point, $\{x^* = 0,\ y^* = 0,\ z^* = 0\}$) is unstable. n_c can also be derived from Eqs.19 because non-zero fixed point must be positive (x, y, z > 0) if it exists in real biological system.

A three-stage model of gene expression

Here we overview a model of gene expression to illustrate the dynamics of a single gene system in our modelling scheme (Fig S2A). This is the basis for our second model of a community effect in animal development.

■ Model description

In this model depicted in Fig S2A, a transcriptional activator, Tf, binds to the promoter of a gene, G, with the rate α_1 and activates its transcription. Tf dissociates from gene G's promoter at the rate α_2 and the gene G returns to inactive state. Activated gene (G_{Tf}) is transcribed to produce mRNA (R) at the rate β, followed by translation to produce protein (P) at the rate γ. The degradation of transcription factors is not considered here, but will be introduced in the complete community effect model described later. mRNAs and translated proteins are degraded at the rate of δ_m and δ_p, respectively.

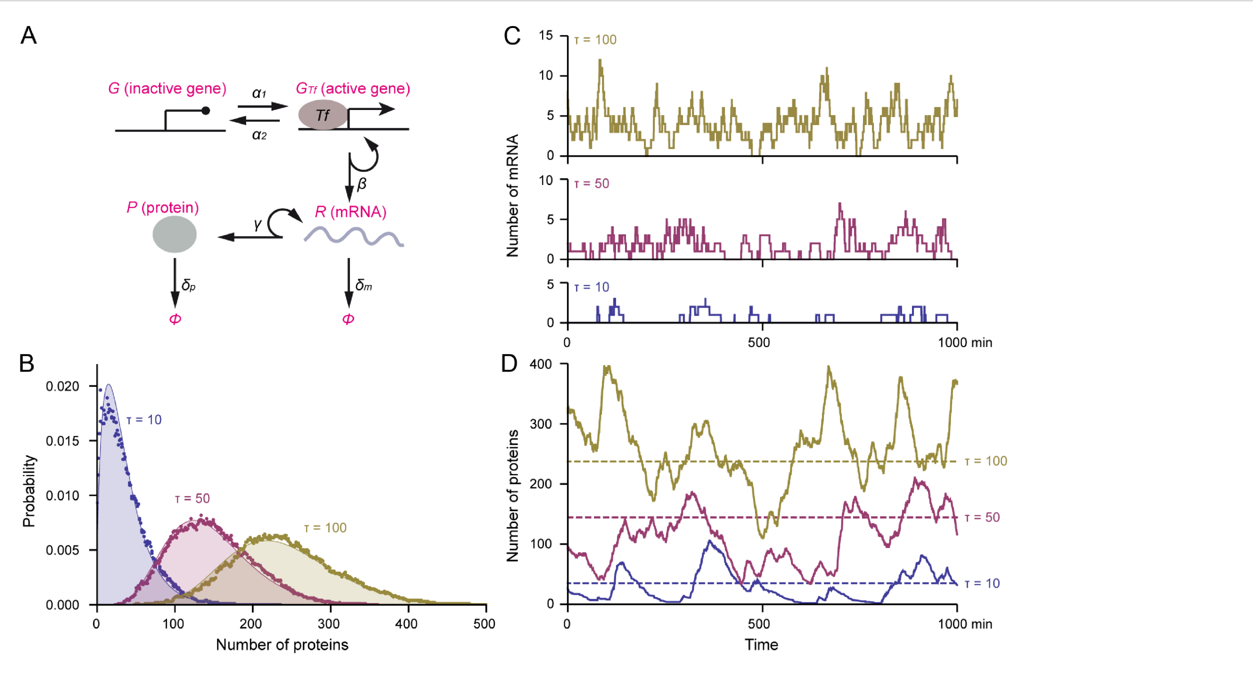

Fig. S2 A three-stage model of gene expression
(A) Diagram of the model. The model consists of inactive gene G, activated gene G_{Tf} (bound by a transcription factor Tf), its transcript R, and the protein product P. 'ϕ' stands for degradation or decay of molecules. Transitions between these states are depicted as arrows with rate constants as indicated (see Table 1 for parameter values).
(B) Probability distributions of protein numbers at steady state. Results of stochastic simulations (dots) and analytical distributions (curves, which are derived from Eq.33) for τ (number of Tf molecules) = 10, 50, 100 are shown.
(C, D) Typical time series of R (mRNA) and P (protein) for τ = 10, 50, 100. Means of protein distribution at steady state are indicated as dotted lines in (D).

The model can be formulated as a set of ordinary differential equations (ODEs) of first-order reactions:

$$[G_{Tf}]' = \alpha_1 [Tf][G] - \alpha_2 [G_{Tf}] \tag{30}$$

$$[R]' = \beta [G_{Tf}] - \delta_m [R] \tag{31}$$

$$[P]' = \gamma [R] - \delta_p [P] \tag{32}$$

Here, the variables in square brackets are continuous numbers of molecules (per unit volume). The gene number is conserved, i.e., $[G] + [G_{Tf}] = 1$. The total number of transcription factor Tf is fixed in the model so that $[Tf] + [G_{Tf}] = \tau$ (constant). When $\tau \gg 1$, $[Tf] \approx \tau$, and the effective gene activation rate constant is given by $\alpha_1 \tau$. Therefore, when $\tau \gg 1$, the model is identical to the three-stage model of gene expression described by Shahrezaei and Swain [11].

Stochastic simulation of the three-stage model of gene expression

Next we show the profile of a single gene expression in our model, which underlies the community effect model described later. We performed stochastic simulations of the single gene model in Fig. S2A for the total number of transcription factor $\tau = 10$, 50 and 100 (Fig. S2C and D). With the parameter values used in our simulations (Table 1), the protein distribution at steady state matches well with the probability distribution of protein numbers P_N described in [11] (negative binomial distribution; Fig. S2B),

$$P_N = \frac{\Gamma(u+N)}{\Gamma(1+N)\,\Gamma(u)} \left(\frac{v}{1+v}\right)^N \left(1 - \frac{v}{1+v}\right)^u \tag{33}$$

Here, Γ is the gamma function and N is the number of proteins at steady state, with $u = \frac{\alpha_1 \tau \beta}{(\alpha_1 \tau + \alpha_2)\,\delta_p}$ and $v = \frac{\gamma}{\delta_m}$. When $\tau \gg 1$, the protein number at steady state in the deterministic model (Eqs.30~32) approaches uv, which is equal to the mean of N, $\langle N \rangle$, in the probability distribution P_N. For $\tau = 10$, 50 and 100 with the parameter set used for simulations (Table 1), the calculated values are $\langle N \rangle \approx 35$, 145 and 238, respectively (dotted lines in Fig. S2D). They are in good agreement with the means of the protein number at steady state in the stochastic simulations, $\langle N \rangle \approx 37$, 148 and 240.

Analysis of the community effect model with transcription

Fixed points

Fixed points (steady state) of Eqs.7~14 in the main text can be solved algebraically. One of the fixed points is trivial:

$$\{[Ag]^* = 1,\ [Ac]^* = 0,\ [Ar]^* = 0,\ [Ap]^* = 0,\ [Bg]^* = 1,\ [Ba]^* = 0,\ [Br]^* = 0,\ [Bp_{in}]^* = 0,\ [Bp_{out}]^* = 0,\ [Cp]^* = 0\} \tag{34}$$

(superscript of asterisk denotes fixed points). This corresponds to cells with no gene expressions. The other fixed point is

$$\begin{aligned}
\Big\{[Ag]^* &= \frac{\alpha_2 \delta_c \delta_m (n\epsilon + \delta_d)(\kappa + \delta_b)(\alpha_1 \beta\gamma + \alpha_2 \delta_a \delta_m)}{\alpha_1 \beta\gamma\,(n\alpha_1 \beta\gamma\epsilon\kappa + \alpha_2 \delta_c \delta_m (n\epsilon + \delta_d)(\kappa + \delta_b))},\\
[Ac]^* &= \frac{n\alpha_1^2 \beta^2 \gamma^2 \epsilon\kappa - \alpha_2^2 \delta_a \delta_c \delta_m^2 (n\epsilon + \delta_d)(\kappa + \delta_b)}{\alpha_1 \beta\gamma\,(n\alpha_1 \beta\gamma\epsilon\kappa + \alpha_2 \delta_c \delta_m (n\epsilon + \delta_d)(\kappa + \delta_b))},\\
[Ar]^* &= \frac{n\alpha_1^2 \beta^2 \gamma^2 \epsilon\kappa - \alpha_2^2 \delta_a \delta_c \delta_m^2 (n\epsilon + \delta_d)(\kappa + \delta_b)}{\alpha_1 \gamma\,\delta_m (n\alpha_1 \beta\gamma\epsilon\kappa + \alpha_2 \delta_c \delta_m (n\epsilon + \delta_d)(\kappa + \delta_b))},\\
[Ap]^* &= \frac{n\alpha_1^2 \beta^2 \gamma^2 \epsilon\kappa - \alpha_2^2 \delta_a \delta_c \delta_m^2 (n\epsilon + \delta_d)(\kappa + \delta_b)}{\alpha_1 \delta_a \delta_m (n\alpha_1 \beta\gamma\epsilon\kappa + \alpha_2 \delta_c \delta_m (n\epsilon + \delta_d)(\kappa + \delta_b))},\\
[Bg]^* &= \frac{\alpha_2 \delta_a \delta_m (n\alpha_1 \beta\gamma\epsilon\kappa + \alpha_2 \delta_c \delta_m (n\epsilon + \delta_d)(\kappa + \delta_b))}{n\alpha_1 \beta\gamma\epsilon\kappa(\alpha_1 \beta\gamma + \alpha_2 \delta_a \delta_m)},\\
[Ba]^* &= \frac{n\alpha_1^2 \beta^2 \gamma^2 \epsilon\kappa - \alpha_2^2 \delta_a \delta_c \delta_m^2 (n\epsilon + \delta_d)(\kappa + \delta_b)}{n\alpha_1 \beta\gamma\epsilon\kappa\,(\alpha_1 \beta\gamma + \alpha_2 \delta_a \delta_m)},\\
[Br]^* &= \frac{n\alpha_1^2 \beta^2 \gamma^2 \epsilon\kappa - \alpha_2^2 \delta_a \delta_c \delta_m^2 (n\epsilon + \delta_d)(\kappa + \delta_b)}{n\alpha_1 \gamma\epsilon\kappa\delta_m (\alpha_1 \beta\gamma + \alpha_2 \delta_a \delta_m)},\\
[Bp_{in}]^* &= \frac{n\alpha_1^2 \beta^2 \gamma^2 \epsilon\kappa - \alpha_2^2 \delta_a \delta_c \delta_m^2 (n\epsilon + \delta_d)(\kappa + \delta_b)}{n\alpha_1 \epsilon\kappa\delta_m (\kappa + \delta_b)(\alpha_1 \beta\gamma + \alpha_2 \delta_a \delta_m)},\\
[Bp_{out}]^* &= \frac{n\alpha_1^2 \beta^2 \gamma^2 \epsilon\kappa - \alpha_2^2 \delta_a \delta_c \delta_m^2 (n\epsilon + \delta_d)(\kappa + \delta_b)}{\alpha_1 \epsilon\delta_m (n\epsilon + \delta_d)(\kappa + \delta_b)(\alpha_1 \beta\gamma + \alpha_2 \delta_a \delta_m)},\\
[Cp]^* &= \frac{n\alpha_1^2 \beta^2 \gamma^2 \epsilon\kappa - \alpha_2^2 \delta_a \delta_c \delta_m^2 (n\epsilon + \delta_d)(\kappa + \delta_b)}{\alpha_1 \delta_c \delta_m (n\epsilon + \delta_d)(\kappa + \delta_b)(\alpha_1 \beta\gamma + \alpha_2 \delta_a \delta_m)}\Big\}.
\end{aligned} \tag{35}$$

■ Derivation of the critical community size n_c

Note that the numerator of all of the solutions in Eqs.35 are identical except $[Ag]^*$ and $[Bg]^*$ (which are both positive numbers), and the denominators are all positive. The condition for all of these values being positive is

$$n\,\alpha_1^2\,\beta^2\,\gamma^2\,\epsilon\,\kappa\;-\;\alpha_2^2\,\delta_a\,\delta_c\,\delta_m^2(n\,\epsilon+\delta_d)\,(\kappa+\delta_b)\;>\;0 \tag{36}$$

Therefore, the condition for the community effect with n cells is

$$n>\frac{\alpha_2^2\,\delta_a\,\delta_c\,\delta_d\,\delta_m^2\,(\kappa+\delta_b)}{\epsilon\,(\alpha_1^2\,\beta^2\,\gamma^2\,\kappa\;-\;\alpha_2^2\,\delta_a\,\delta_c\,\delta_m^2\,(\kappa+\delta_b))}=n_c, \tag{37}$$

which leads to Eqs.15 and 16 in the main text.

In more general case when multiple copies of genes are present per cell, such as diploid cells, the solution for n_c is a modified form of Eq.37. Let a and b be the copy numbers of Gene A and Gene B, respectively, i.e., $[Ag]+[Ac]=a$ and $[Bg]+[Ba]=b$. n_c can be derived in a similar manner as above:

$$n_c=\frac{\alpha_2^2\,\delta_a\,\delta_c\,\delta_d\,\delta_m^2\,(\kappa+\delta_b)}{\epsilon\,(a\,b\,\alpha_1^2\,\beta^2\,\gamma^2\,\kappa\;-\;\alpha_2^2\,\delta_a\,\delta_c\,\delta_m^2\,(\kappa+\delta_b))} \tag{38}$$

which yields Eq.17 in the main text.

■ Theoretical limit of $[Ap]$

According to Eqs.35, as n increases, $[Ap]$ at steady state ($[Ap]^*$) approaches

$$[Ap]^*_{n\to\infty}=\frac{\alpha_1^2\,\beta^2\,\gamma^2\,\kappa\;-\;\alpha_2^2\,\delta_a\,\delta_c\,\delta_m^2\,(\kappa+\delta_b)}{\alpha_1\,\delta_a\,\delta_m\,(\alpha_1\,\beta\,\gamma\,\kappa\;+\;\alpha_2\,\delta_c\,\delta_m(\kappa+\delta_b))}\,. \tag{39}$$

Interestingly, this is identical to $[Ap]^*_{\epsilon\to\infty}$ and $[Ap]^*_{\delta_d\to 0}$, and is also independent of ϵ and δ_d. Therefore, the theoretical maximum of $[Ap]^*$ ($[Ap]^*_{\max}$) is independent of the process outside the cell. With the parameter values in Table 1 and $a=1$, $b=1$, $[Ap]^*_{\max}\simeq 379$. This is distinct from the maximum capacity of gene A's expression, which is $\frac{\beta\gamma}{\delta_a\,\delta_m}$ ($\simeq$ 666 with parameter values in Table 1).

A model of diffusion in a spherical tissue

In this section, we argue our mean-field approximation for a community effect is justified. We consider a spherical tissue with radius $R2$ that contains a spherical cell group of radius $R1$ ($< R2$). The inner cell group produces and releases a diffusible factor into the extracellular space at the constant rate v, which diffuses in the entire tissue and is degraded or taken up by cells at the rate δ uniformly in the tissue sphere. The inner tissue can be regarded as a cell group responsive to a community effect. The surrounding tissue ($R1 < r \leq R2$; r is a distance from the center of the concentric sphere) does not produce the diffusible molecule. Such system is described by a diffusion equation,

$$\frac{\partial c}{\partial t} = D \nabla^2 c - \delta c + v \theta \tag{40}$$

where $\theta = 1$ for $0 \leq r \leq R1$ and 0 for $R1 < r \leq R2$. c is the concentration of the diffusible factor, D is the diffusion coefficient and ∇^2 is the Laplacian for spherical coordinates. The production term v in Eq.40 can be substituted by $\frac{v c}{c+k}$ that reflects a community effect but introduces another parameter k into the equation. Numerical simulations of such model have shown qualitatively similar characteristics to the model without this modification. Because it introduces k and does not alter the following argument, we have not analysed it in more detail. Because of radial symmetry, Eq.40 is reduced to

$$\frac{\partial c}{\partial t} = D\left(\frac{\partial^2 c}{\partial r^2} + \frac{2}{r}\frac{\partial c}{\partial r}\right) - \delta c + v \theta. \tag{41}$$

The tissue sphere is treated as a closed system, therefore, the boundary condition at $r = R2$ *is*

$$\left.\frac{\partial c}{\partial r}\right|_{r=R2} = 0. \tag{42}$$

Likewise, because the tissue is symmetrical about the center $r = 0$,

$$\left.\frac{\partial c}{\partial r}\right|_{r=0} = 0. \tag{43}$$

The initial condition is set to $c_{t=0} > 0$ for all r, that is, c is uniform at t = 0. The steady state solution for Eq.41 is independent of the initial condition and described as

$$\begin{aligned} c^i(r) &= \frac{a_1 e^{-r\sqrt{\frac{\delta}{D}}}}{r} + \frac{a_2 e^{r\sqrt{\frac{\delta}{D}}}}{r} + \frac{v}{\delta} \\ c^o(r) &= \frac{a_3 e^{-r\sqrt{\frac{\delta}{D}}}}{r} + \frac{a_4 e^{r\sqrt{\frac{\delta}{D}}}}{r} \end{aligned} \tag{44}$$

where $c^i(r)$ is the concentration of diffusible factors in the inner tissue ($0 \leq r \leq R1$), $c^o(r)$ for the outer tissue ($R1 < r \leq R2$) and a_1, a_2, a_3, a_4 are constants. From $c^i(0) \neq \infty$ and Eq.43, $a_1 + a_2 = 0$. Using Eq.42 and the other conditions at the steady state,

$$c^i(R1) = c^o(R1) \tag{45}$$

$$\left.\frac{d c^i(r)}{d r}\right|_{r=R1} = \left.\frac{d c^o(r)}{d r}\right|_{r=R1}, \tag{46}$$

the constants $a_1 \sim a_4$ can be solved analytically, which are the functions of parameters $R1$, $R2$, D, v and δ (the solution is not shown because of its length and complexity).

Yu *et al*. measured the diffusion coefficient of the FGF8 protein (a homologue of FGF4, M.W. = 22 *kDa*) labelled with the organic dye Cy5 (M.W. = 1 *kDa*) to be ~$91 \mu m^2/s$ in living zebrafish embryos and concluded that FGF8 proteins diffuse freely in the extracellular space [16]. They also estimated its half-life in the extracellular space to be about 18 min, which corresponds to $\delta = 6.4 \times 10^{-4}$. Assuming that a similar physiological condition applies to *Xenopus* embryos, we adopted these values to analyse our diffusion model. Fig. S3 shows how the gradient profile of c in the tissue evolves over time. The concentration of the diffusible factor ($R1 = 400 \mu m$, $R2 = 500 \mu m$, $D = 0.1 \mu m^2/s$ in panel A and $100 \mu m^2/s$ in B) is normalized by the inner sphere's production capacity defined by v/δ. The normalized concentration c_n takes a value between 0 and 1. With this range of diffusion constant that is relevant to the community effect in the embryos, the gradient profile of c^i looks fairly flat at any moment.

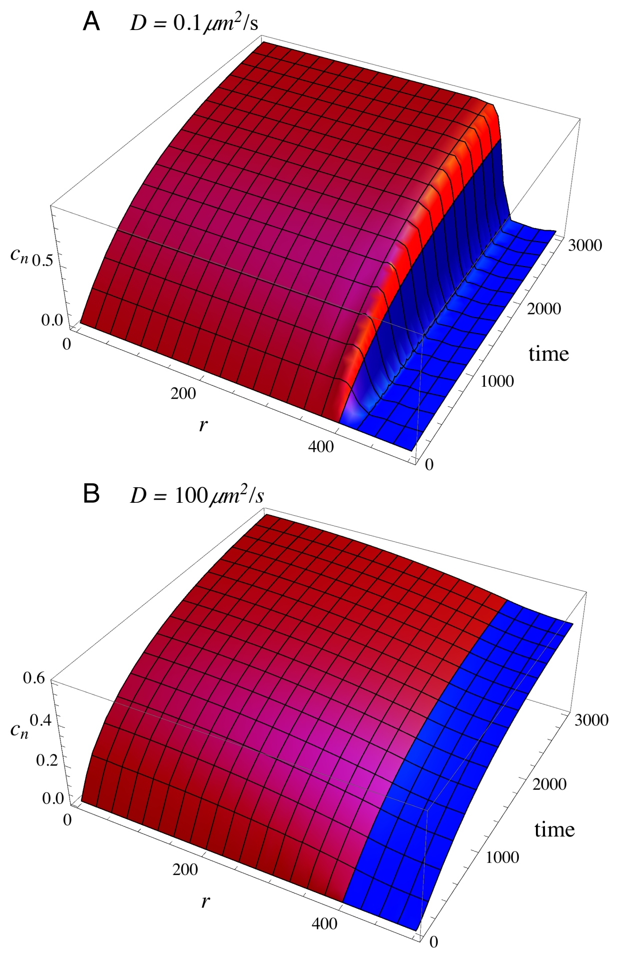

Fig. S3 Concentration change over time across the tissue.
Red: inner tissue sphere; blue: outer tissue layer. For these numerical simulations, $c_{t=0} = 50$. Values of D are as indicated for each panel. $R1 = 400\mu$m, $R2 = 500\mu$m, $\upsilon = 10$, $\delta = 0.001$.

As another way to quantify how steep is the 'gradient' of $c^i(r)$ across the inner tissue sphere, we introduce the quantity ϕ that is defined as

$$\phi = \frac{c^i(0) - c^i(R1)}{c^i(0)}. \tag{47}$$

Smaller ϕ means less steep gradient profile. $c^i(0)$ can be solved as

$$\begin{aligned} c^i(0) &= \frac{\upsilon}{\delta} - 2\,a_1\sqrt{\frac{\delta}{D}} \\ &= \frac{\upsilon}{\delta} - \psi(R1, R2, D, \upsilon, \delta). \end{aligned} \tag{48}$$

where ψ is a certain function of $R1$, $R2$, D, υ and δ. From Eqs.44 and 48, ϕ can be obtained as a function of $R1$, $R2$, D and δ, and is actually independent of the production rate υ. We have examined how ϕ is altered by the parameters $R1$ (radius of inner tissue) and δ (decay rate of diffusive factor). Fig. S4 is a density plot of ϕ as a function of $R1$ and δ, with $D = 100\mu\text{m}^2$/s.

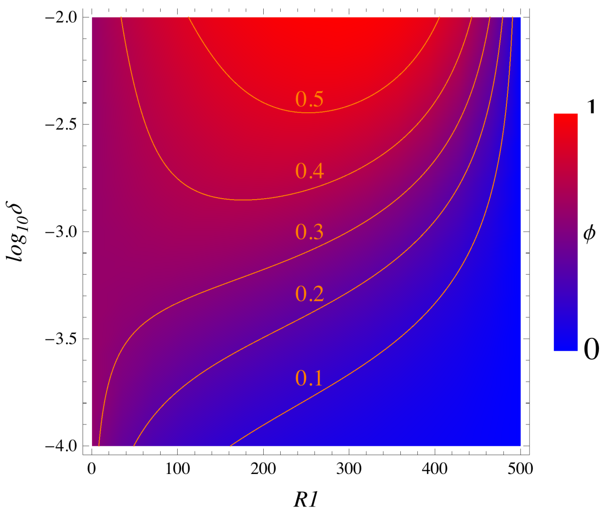

Fig. S4 The ‘gradient’ of $c^i(r)$ across the inner tissue sphere
D = 100μm^2/s is used for this plot. Orange lines are contours with values as indicated.

For $R1$ = 400μm, $R2$ = 500μm and δ = 0.001 (half-life ~ 12 min), $\phi \approx 0.18$. From this plot one can conclude that, in general, the larger the inner tissue sphere is, the flatter c^i gradient across the tissue is. Similarly, the smaller the decay rate is, the flatter the gradient is. With the same parameter values and the ratio of $R1$ to $R2$ fixed, smaller $R2$ (i.e., smaller tissue size) generates less steep c^igradient.

We next examined how the diffusion rate D affects the concentration gradient profile of the diffusible molecule. Fig. S5 shows a gradient profile of c at steady state across the tissue ($0 \le r \le R2$), plotted as a function of D. With a small diffusion rate close to D = 0.1μm^2/s, c^i gradient is almost flat across the inner tissue sphere and the concentration is close to the maxima (c_n = 1). As D becomes larger, the proportion of inner tissue that is exposed to the highest concentration of diffusible factor, i.e., $c^i(0)$, decreases. Also in general, c_n decreases as D increases within the range examined.

We have therefore concluded that, within the relevant range and right combination of parameters, a large proportion of the inner tissue is exposed to a similar concentration of diffusible factors. These analyses justify the mean-field simplification in our community effect model, which does not take the diffusion of extracellular factors (Bp_{out}) into account.

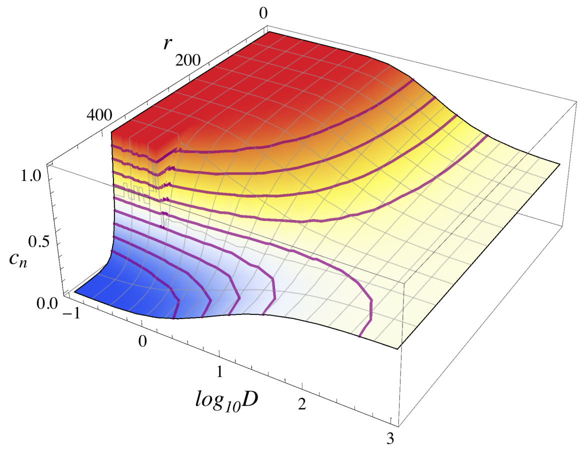

Fig. S5 The steady-state profile of c_n across the tissue plotted as a function of diffusion rate D
The parameter values for this plot is: $R1$ = 400μm, $R2$ = 500μm, υ = 10, δ = 0.001. The purple lines are contours of c_n = 0.1 m, m=1, 2, ..., 9.
